# Intratracheal instillation of pravastatin for the treatment of murine allergic asthma: a lung-targeted approach to deliver statins

**DOI:** 10.14814/phy2.12352

**Published:** 2015-05-11

**Authors:** Amir A Zeki, Jennifer M Bratt, Kevin Y Chang, Lisa M Franzi, Sean Ott, Mark Silveria, Oliver Fiehn, Jerold A Last, Nicholas J Kenyon

**Affiliations:** 1 University of CaliforniaDavis, California; 2 Department of Internal Medicine, University of CaliforniaDavis, California; 3 Division of Pulmonary, Critical Care and Sleep Medicine, University of CaliforniaDavis, California; 4 Center for Comparative Respiratory Biology and Medicine (CCRBM), University of CaliforniaDavis, California; 5 U.C. Davis, West Coast Metabolomics Center (WCMC), University of CaliforniaDavis, California; 6 King Abdulaziz University, Biochemistry DepartmentJeddah, Saudi Arabia

**Keywords:** Airway hyperreactivity, airway hypersensitivity, airway inflammation, asthma, asthma treatment, goblet cells, inhaled statin, intratracheal statin, mass spectrometry, pravastatin, remodeling

## Abstract

Systemic treatment with statins mitigates allergic airway inflammation, T_H_2 cytokine production, epithelial mucus production, and airway hyperreactivity (AHR) in murine models of asthma. *We hypothesized that* pravastatin delivered intratracheally would be quantifiable in lung tissues using mass spectrometry, achieve high drug concentrations in the lung with minimal systemic absorption, and mitigate airway inflammation and structural changes induced by ovalbumin. Male BALB/c mice were sensitized to ovalbumin (OVA) over 4 weeks, then exposed to 1% OVA aerosol or filtered air (FA) over 2 weeks. Mice received intratracheal instillations of pravastatin before and after each OVA exposure (30 mg/kg). Ultra performance liquid chromatography – mass spectrometry was used to quantify plasma, lung, and bronchoalveolar lavage fluid (BALF) pravastatin concentration. Pravastatin was quantifiable in mouse plasma, lung tissue, and BALF (BALF > lung > plasma for OVA and FA groups). At these concentrations pravastatin inhibited airway goblet cell hyperplasia/metaplasia, and reduced BALF levels of cytokines TNF*α* and KC, but did not reduce BALF total leukocyte or eosinophil cell counts. While pravastatin did not mitigate AHR, it did inhibit airway hypersensitivity (AHS). In this proof-of-principle study, using novel mass spectrometry methods we show that pravastatin is quantifiable in tissues, achieves high levels in mouse lungs with minimal systemic absorption, and mitigates some pathological features of allergic asthma. Inhaled pravastatin may be beneficial for the treatment of asthma by having direct airway effects independent of a potent anti-inflammatory effect. Statins with greater lipophilicity may achieve better anti-inflammatory effects warranting further research.

## Introduction

Asthma affects 27 million Americans, the World Health Organization estimates that 235 million people have asthma worldwide, and the prevalence continues to rise. Investigators are actively exploring new treatments for asthma, in particular novel inhaler therapies. We and others have previously shown that systemic treatment with statins in animal models of asthma (Zeki et al. 2009) and chronic obstructive pulmonary disease (COPD) (Davis et al. 2013) mitigates inflammation and early features of airway remodeling (Zeki et al. 2010). In this current work, we explore whether delivering statins directly to the lungs by intratracheal (i.t.) instillation can mitigate experimental asthma.

The statin drugs (‘statins’), which inhibit the 3-hydroxy-3-methyl-glutaryl-CoA reductase (HMGCR) enzyme, are highly effective medications for the treatment of atherosclerosis and cardiovascular diseases. Statins inhibit HMGCR, the rate-limiting step of cholesterol biosynthesis in the mevalonate (MA) cascade. Statins also exhibit pleiotropic properties that result in a wide range of cellular and physiological effects by both HMGCR-dependent and -independent mechanisms (Wang et al. 2008; Zeki et al. 2011). They have also garnered wide interest giving rise to studies investigating their therapeutic benefits in different diseases including lung diseases such as asthma, COPD, pulmonary hypertension, lung cancer, pneumonia, and bronchiectasis.

In both cell culture experiments and animal models, systemic administration of the statins (including simvastatin, lovastatin, pravastatin, and atorvastatin) manifests beneficial anti-inflammatory (Kim et al. 2007; Imamura et al. 2009), anti-proliferative (Takeda et al. 2006; Capra and Rovati 2014), anti-fibrotic (Watts et al. 2005), anti-oxidant (Mctaggart 2003; Shishehbor et al. 2003; Melo et al. 2013), and immunomodulatory properties (Greenwood et al. 2006; Morimoto et al. 2006). In preclinical models of asthma, systemic administration of statins (i.e., oral and/or intraperitoneal) inhibits allergic inflammation (Zeki et al. 2009), AHR, goblet cell metaplasia/hyperplasia, and profibrotic changes in airways (McKay et al. 2004; Yeh and Huang 2004; Chiba et al. 2008a; Ou et al. 2008; Ahmad et al. 2011; Huang et al. 2013).

Human observational studies in patients with asthma indicate a positive correlation between improved lung function, fewer exacerbations, and reduced corticosteroid use in statin users versus nonusers (Alexeeff et al. 2007; Huang et al. 2011; Tse et al. 2013, 2014). However, several small clinical trials in asthma have not confirmed such statin benefits despite showing reduced sputum inflammatory cytokine levels and cell counts, and improvement in quality of life survey scores (Hothersall et al. 2008; Fahimi et al. 2009; Cowan et al. 2010; Maneechotesuwan et al. 2010; Braganza et al. 2011; Moini et al. 2012). Despite the limitations of these randomized clinical trials (RCTs) (e.g., short treatment durations, lack of outcome data such as severe exacerbations) interest remains in the potential beneficial effect of statins in those with severe asthma (Zeki et al. 2013).

The statins used in these RCTs were given to study subjects via the oral route which is the only approved route of statin administration in humans. It remains unknown whether the statins used (simvastatin, atorvastatin) reached the airway compartment or whether these statins target the lung directly or indirectly through peripheral immune mechanisms. Despite the lung being the target organ, it has not been determined whether orally ingested statins penetrate into human lungs or airways. Also, it remains unclear what the relative effects of statins are in different anatomic compartments, in particular, the blood, lung parenchyema, and airways. Therefore, it may be beneficial to give statins via inhalation to directly target the airway epithelium, a central player in asthma pathogenesis. The potential advantage of this approach would be the use of lower drug doses than that used systemically, in order to achieve greater local potency in the airways while reducing risk of systemic side effects.

Several in vitro studies using different lung resident cell types have shown statin benefits, including airway smooth muscle and airway epithelial cells (Sakoda et al. 2006; Takeda et al. 2006; Murphy et al. 2008; Schaafsma et al. 2011; Iwata et al. 2012). We have shown that mouse tracheal epithelium may be a viable target for statins (Zeki et al. 2012), where treatment with simvastatin inhibited the expression of key IL13-inducible genes important in helper T-cell type 2 (Th2) inflammation, such as eotaxin-1 and the monocyte chemotactic peptides.

Based on this knowledge, the direct instillation of a statin into mouse trachea in vivo is predicted to inhibit allergen-induced airway inflammation and improve lung function, in a manner comparable to systemic statin treatment (Kim et al. 2007; Chiba et al. 2008a; Imamura et al. 2009; Zeki et al. 2009). Therefore, w*e hypothesized* that intratracheal instillation of a water-soluble statin, pravastatin, would (1) produce quantifiable levels of pravastatin in blood plasma, lung tissue, and bronchoalveolar lavage fluid (BALF) using mass spectrometry; (2) achieve high drug concentrations locally in the lung with minimal systemic absorption; and (3) reduce eosinophilic inflammation, goblet cell hyperplasia, and airway hyperresponsiveness.

In this proof-of-principle study, we report a novel method for quantifying pravastatin in plasma, homogenized lung tissue, and BALF specimens using ultra performance liquid chromatography – mass spectrometry (UPLC-MS), and show that i.t. pravastatin reduces allergen-induced airway goblet cell hyperplasia and airway hypersensitivity (AHS), without significantly affecting AHR or airway inflammation. Pravastatin administered i.t. is well-tolerated and detectible in high concentrations in OVA-exposed mouse lung tissue as compared to plasma. This work suggests that statins may be targeted for delivery to the lung and potentially developed as a novel class of inhaler therapy for the treatment of human asthma.

## Materials and Methods

### Animals

Male BALB/c mice, 8 to 10 weeks old and weighing between 18 to 20 g, were purchased from the Jackson Laboratory (Sacramento, CA), and housed in an AALAC-accredited facility equipped with HEPA-filtered laminar flow cage racks. Mice were maintained on a 12-h light/dark cycle with food and water given ad lib, and were routinely screened for health status by serology and histology by veterinary staff of the Animal Resource Services. Animals were housed no more than four mice per cage, and mice were allowed to acclimate for 1 week prior to starting experiments. All procedures were performed under an IACUC-approved protocol at the University of California, Davis.

### Sensitization and exposure of mice to ovalbumin and treatment with pravastatin

Mice were sensitized by two intraperitoneal (i.p.) injections of 10 *μ*g/0.1 mL chicken egg albumin (i.e., ovalbumin (OVA), grade V, ≥98%; Sigma, St. Louis) with alum adjuvant on days 1 and 14 of the protocol (Temelkovski et al. 1998). The mice were divided into four groups; OVA+ i.t. PBS (*n* = 16), OVA + i.t. Prav (*n* = 15), FA + i.t PBS (*n* = 12), and FA + i.t. Prav (*n* = 14). Starting on day 28, mice were exposed to either an aerosol of 1% OVA dissolved in PBS (8 mL) for 30 min or filtered air. Aerosols were generated by a Hudson RCI side-stream nebulizer (Teleflex; Research Triangle Park, NJ) and Passport Compressor (Invacare; Sanford, FL). Exposure was repeated three times per week for 2 weeks (for a total of six exposures).

Pravastatin (Cayman Chemical; Ann Arbor, MI) was dissolved in Dulbecco's phosphate-buffered saline (PBS) at a concentration of 9 mg/mL (pH 7.45). Thirty minutes prior to and after each OVA exposure, mice were briefly anesthetized using Isoflurane (Attane^TM^) administered via inhalation in an enclosed chamber. Once anesthetized, they were placed in a supine position, then the tongue and bottom jaw were gently drawn open. A Hamilton glass syringe (Model #1705, Hamilton; Reno, NV) with a blunt-tipped 22-gauge needle was loaded with 50 *μ*L volume of pravastatin solution. Tracheal insertion was confirmed by palpation of the tracheal rings with the needle tip during insertion (Wegesser and Last 2009). Pravastatin (15 mg/kg dose per instillation) was instilled into the trachea (i.e., intratracheal (i.t.)) as a single dose 30 min prior to *and* after each OVA exposure to achieve target total dose of 30 mg/kg (see below). Animals were allowed to recover in an upright position until ambulatory before being placed back into their cages.

### Lung physiology and exhaled nitric oxide measurements

After the completion of the final OVA aerosol or FA exposure, mice were deeply anesthetized with medetomidine (Domitor 0.75 mg/kg, Orion Pharma; Finland) and tiletamine/zolpidem (Telazol 37.5 mg/kg; Fort Dodge Laboratories; Fort Dodge, IA). Mice were cannulated and ventilated at 8 mL/kg using a mouse ventilator (MiniVent, Harvard Apparatus; Cambridge, MA) in a whole body plethysmograph for restrained animals (Buxco, Inc.; Troy, NY). A 5-min sample of exhaled breath was collected in Mylar bags via the exhalation port of the ventilator during baseline lung physiology measurements, and the fraction of exhaled nitric oxide (FeNO) was measured in parts per billion (ppb) by a chemiluminescence assay using the Sievers Nitric Oxide Analyzer (GE Instruments, Sievers; Boulder, CO). Placement of the Mylar bag did not affect pressure measurements.

Lung physiology (dynamic compliance (C_dyn_) and respiratory system resistance (*R*_rs_)) was measured by plethysmograph using the BioSystem XA software (Buxco, Inc., Troy, NY). These parameters were measured in 10 sec increments and averaged over a 5-min baseline period, a 3-min saline aerosol exposure period, and a series of 3-min methacholine (MCh) aerosol dose challenges (0, 0.5, 1.0, and 2.0 mg/mL) (Kenyon et al. 2003) to determine airway hyperreactivity (AHR) and airway hypersensitivity (AHS).

AHR is defined as greater airway reactivity to a given dose of MCh or over increasing doses of MCh represented by a steeper slope of the dose–response curve; and AHS is defined as increased airway resistance above baseline occurring at a lower effective dose of MCh than its respective control (Affonce and Lutchen 2006), that is, the airways require less contractile stimulus when compared with healthy airways. We use the term “airway hyperresponsiveness” to include both AHR and AHS (O'Byrne and Inman 2003; Turi et al. 2011).

### Blood collection

Mice were killed with an overdose of Beuthanasia-D (pentobarbital sodium and phenytoin sodium) by intraperitoneal (i.p.) injection at the conclusion of lung physiology measurements. Blood was collected via cardiac puncture in heparinized tubes (~1 mL). Plasma was obtained by centrifugation at 4°C and stored at −80°C for further measurement of pravastatin by UPLC-MS.

### Bronchoalveolar lavage fluid (BALF) collection and inflammatory cells counts

Mice tracheas were cannulated; and lungs were lavaged using two 1 mL aliquots of sterile PBS (pH 7.4) containing a general protease inhibitor cocktail with aprotinin and leupeptin (1:100; Sigma; St. Louis, MO) and 0.1 *μ*mol/L phenylmethanesulfonylfluoride (PMSF), with each aliquot passaged twice. The collected BALF was centrifuged at 2000 rpm on a bench top centrifuge and the resulting supernatant was decanted and stored at −80°C for cytokine analysis and UPLC-MS measurement of pravastatin. The remaining cell pellet was resuspended in ACK lysis buffer (0.15 mol/L NH_4_Cl, 1 mmol/L KHCO_3_, 0.1 mmol/L EDTA, pH 7.3), then centrifuged for an additional 10 min. The subsequent cell pellet was re-suspended in 0.5 mL PBS, and an aliquot of this fluid was removed for live cell counting.

A 20 *μ*L volume of the cell suspension was used to determine the BALF total live cell counts using a hemacytometer and Trypan Blue exclusion method. Then a 100 *μ*L volume of the cell suspension was also processed onto slides using a cytofuge. Slides were air-dried then stained with a Hema3 stain set per the manufacturer's instructions (Fisher Scientific; Kalamazoo, MI). Cell percent differentials were calculated by counting 10 fields at 400× and classifying cell types as macrophage, neutrophil, eosinophil, lymphocyte, or “other” based upon standard morphological characteristics and staining profile.

### Bronchoalveolar lavage cytokine levels

The concentrations of selected helper T-cell type 1 (Th1), Th2, and Th17 cytokines and chemokines from BALF supernatant were measured with commercially available multiplex assays (EMD Millipore; Billerica, MA). For cytokine/chemokine sample measurements below the lower detection limit, results were assigned a value equal to the minimal detection limit for the specific assay to facilitate statistical analysis of the data.

### Lung histopathology

After completion of lung lavage, the chest cavity was exposed and the right bronchus was ligated using surgical suture. The right lung lobes were isolated and snap frozen on dry ice for UPLC-MS measurements. The remaining intact left lung lobe was fixed for histological examination in situ under constant hydrostatic pressure of 30 cm H_2_O using 1% paraformaldehyde (PFA) in PBS (pH 7.4), and embedded in paraffin blocks for sectioning.

Lung histolopathology was examined for the qualitative assessment of peribronchial inflammation and goblet cell metaplasia/hyperplasia as previously described Zeki et al. (2010). Paraffin-embedded left lung lobes were sectioned at a thickness of 5 microns parallel to the main conducting airways. Sections were stained with either Alcian Blue-Periodic acid Schiff (PAS) or with hematoxylin and eosin (H&E) to assess the degree of goblet cell hyperplasia/metaplasia or peribronchial and perivascular inflammation, respectively.

Each animal was represented by a single section of lung that included the 2^nd^, 3^rd^, and 4^th^ generations of conducting airways. Five randomly selected regions were evaluated, including 2 segments of the 2^nd^ generation, 2 segments from the 3^rd^ generation, and 1 segment from a 4^th^ generation of conducting airways. A minimum of 100 sequential airway epithelial cells were counted from each region, and the ratio of PAS positive cells per total epithelial cells was determined for each region. These regional values then were averaged to give a final PAS score per animal reported as “% Positive PAS Cells”.

### Mass spectrometry to measure pravastatin

Blood plasma, BALF, and lung samples were used to determine pravastatin tissue absorption. Blood and BALF specimens were processed as described above. Right lung samples were homogenized on ice using a mixture of stainless steel and silica beads in a 2010 Geno/Grinder (Spex SamplePrep; Metuchen, NJ) at 1500 strokes/min.

As part of our method development, the optimal mixture was determined to be an acetonitrile-H_2_O solution (1:1, v/v), as compared to isopropanol/acetonitrile/water or methanol/water. This acetonitrile solution was used to extract pravastatin from specimens of plasma, BALF, and whole lung homogenates. Samples were vortexed and centrifuged, the supernatant was transferred to a new tube and dried completely using a Labconco Centrivap Speedvac, then resuspended in 100 *μ*L of acetonitrile-H_2_O solution (1:1, v/v). Sample supernatants were analyzed by ultra performance liquid chromatography – mass spectrometry (UPLC-MS). Separation was performed by a Waters Acuity UPLC on a Waters HSS T3 reversed phase 100 Å, 1.8 *μ*m, 2.1 mm × 30 mm column. A 6 min gradient (at 0.4 mL/min) of 10 mmol/L ammonium formate with 0.1% formic acid (A) and acetonitrile with 0.1% formic acid (B) was formed by starting with 30% B, increasing to 95% B at 4 min, followed by 2 min of equilibration.

An AB Sciex Qtrap 4000 mass spectrometer was set to monitor the most optimum transition for Pravastatin precursor-to-product of m/z 423.2 ([M-H]-) → m/z 100.8 using MRM (Multiple Reaction Monitoring) in negative mode. Quantitation was performed for this transition using a standard curve made from known stock solutions. Optimized conditions for best sensitivity were found at a collision energy of −47.7 volts and declustering potential of −109 volts at its retention time. The limit of detection (LOD) was determined to be 350 pg/*μ*L.

### Calculating pravastatin concentration in lung tissue

**Figure 1:**
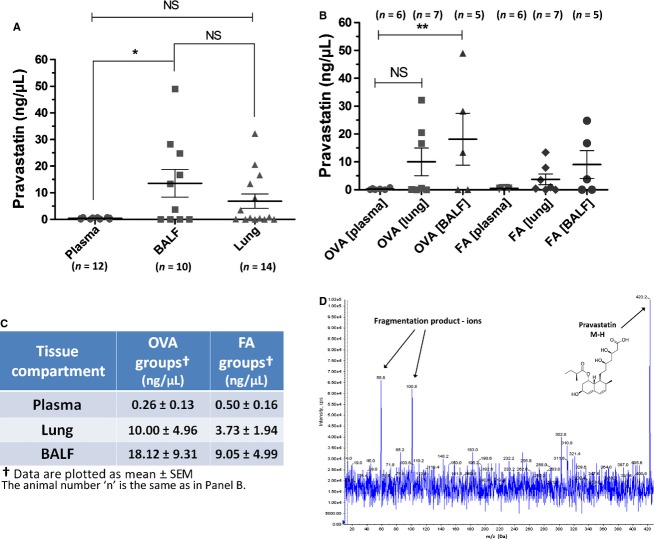
Pravastatin Quantification Using Mass Spectrometry and Determination of Concentrations in Three Tissue Compartments – Plasma, BALF, and Lung. (A) Plasma, BALF, and lung pravastatin concentrations were measured combining mice from all four treatment groups (OVA and FA), plotted by tissue compartment. Pravastatin was not detected in PBS drug vehicle controls. Pravastatin concentration in BALF was 35.7-fold higher than plasma (Plasma 0.38 ± 0.11 vs. BALF 13.58 ± 5.21 ng/*μ*L, *P = 0.0039; and for Lung 6.87 ± 2.70 ng/*μ*L vs. Plasma, or Lung vs. BALF,* P* = NS by Kruskal–Wallis test). (B) In OVA-exposed mice, whereas the mean pravastatin concentration in BALF was 69.7-fold higher than plasma, and in lung was 38.5-fold higher than plasma, only OVA_BALF_ and OVA_plasma_ showed a statistically significant difference in pravastatin concentration (**P = 0.0094 by 1-way ANOVA). All other relevant comparisons were not statistically significant (*P* = NS) (Including OVA_BALF_ vs. FA_BALF_, OVA_lung_ vs. FA_lung_ and OVA_plasma_ vs. FA_plasma_). (C) This panel is the tabular representation of the graph in panel (B). (D) We determined the MS/MS spectrum of pravastatin in both lung tissue and plasma using ultra performance liquid chromatography – mass spectrometry (UPLC-MS), which detected molecular ions in negative ionization mode. This figure shows the spectrum of pravastatin using pure drug standard, where the molecular structure of pravastatin is indicated at the pravastatin peak (right-side black arrow). Two other fragmentation products are indicated with black arrows (left side of figure). *Abberviations:* m/z = mass to charge ratio, Da = Daltons, cps = counts per second. The number of mice (*n*) is indicated in parentheses for each experimental group.

While pravastatin concentrations in nanogram per gram of lung (ng/g) would enable us to calculate absolute values (i.e., exact drug concentrations) for pravastatin in lung tissue, estimating that homogenized mouse lung has a density similar to (or slightly higher than) water at ≥1 g/mL, units of ng/*μ*L allowed us to compare drug levels between plasma, BALF, and lung tissue samples. We recognize the limitations of assuming a value of 1 g/mL density for lung tissue. Variations in density may exist between OVA and FA groups due to factors such as edema, inflammation, and mucin production which cannot be addressed in this calculation without previously determining the lung dry weight, % solubility of the tissue, and final vol/weight ratio of the lung homogenate (Fig.1C). However, it is important to note that while the *absolute* pravastatin levels (i.e., exact drug concentrations) would be different, the *relative* changes in pravastatin partitioning among the different tissue compartments will likely be the same (or similar) to our results using this estimate of homogenized lung density.

### Statistical analysis

Our results represent data from three independent experiments. Data were analyzed using the Prism 5 software package (GraphPad, Inc.; San Diego, CA). All data were tested for normality using the D'Agostino and Pearson omnibus test. Where appropriate data were log or natural log (Ln) transformed and re-assessed for normality, then tested for statistical significance using parametric or nonparametric tests. Where appropriate, data that were two standard deviations (SDs) outside the mean were considered extreme outliers and excluded from analysis. Mice with BALF eosinophil counts < 30% in our OVA model are considered allergen nonresponders; one animal considered a nonresponder was excluded from analysis. Responders to OVA in our model typically have at least ≥60% BALF eosinophilia and on average we observe 70 to 80% eosinophilia. For parametric data, we used the t test or ANOVA with Tukey's posttest correction for 1-way ANOVA and Bonferroni's posttest correction for 2-way ANOVA. For nonparametric data, we used the Mann–Whitney or Kruskal–Walis test with Dunn's posttest correction to determine statistical significance. For unequal variance, Welch's correction for t tests was used where appropriate. The Wilcoxon signed rank test was used to analyze median values with a nonparametric distribution. Data are plotted as means ± SEM except where indicated.

## Results

### Pravastatin concentration in plasma and lung tissues

The first question we addressed was whether pravastatin was measurable in the lung in greater quantities than the systemic circulation following i.t. instillation. To determine this we used UPLC-MS to quantify pravastatin levels in plasma, BALF, and lung tissue homogenates. The detection of pravastatin was most sensitive in the negative ionization mode due to reduced noise levels by coeluting compounds or matrix components. Pravastatin was readily detected by tandem MS/MS fragmentation of the deprotonated pravastatin precursor ion m/z 423.2 with two major fragmentation products at m/z 58.8 and m/z 100.8 (acetate and 2-methylbutanoate substructure fragments, respectively). An example of the MS/MS spectrum of pravastatin is shown in Fig.1D, using pure drug as a standard. Mice administered the vehicle (PBS) alone did not have detectible levels of pravastatin in any of the tissue compartments analyzed, as expected.

Combining the OVA and FA groups and comparing by tissue compartment, measured levels of BALF pravastatin were significantly higher than plasma pravastatin. On average, BALF pravastatin concentration was 35.7-fold higher than plasma (plasma 0.38 ± 0.11 vs. BALF 13.58 ± 5.21 ng/*μ*L, *P = 0.0039 by Kruskal–Wallis test). There was no significant difference between the lung compartment pravastatin concentration (6.87 ± 2.70 ng/*μ*L) and plasma or BALF pravastatin concentration (Fig.1A).

Comparing the OVA vs. FA groups, OVA-exposed mice exhibited the highest mean concentrations of pravastatin in the BALF (18.12 ± 9.31 ng/*μ*L) and lung tissue (10.00 ± 4.96 ng/*μ*L) with minimal pravastatin detected in plasma (0.26 ± 0.13 ng/*μ*L) indicating low systemic absorption (Fig.1B and C). In the OVA groups, mean pravastatin concentration in BALF is 69.7-fold higher than plasma (OVA_BALF_ 18.12 ± 9.31 vs. OVA_plasma_ 0.26 ± 0.13 ng/*μ*L, **P = 0.0094 by 1-way ANOVA), and in lung it is nearly 38.5-fold higher than plasma. FA-exposed mice exhibited similar trends in tissue distribution to their OVA-exposed counterparts, but the differences were not statistically significant. All other relevant comparisons including compartmental comparisons between FA- and OVA-exposed groups were not statistically significant, that is, OVA_BALF_ vs. FA_BALF_, OVA_lung_ vs. FA_lung_, and OVA_plasma_ vs. FA_plasma_ (*P* = NS by 1-way ANOVA, Fig.1B). These results indicate that while the majority of pravastatin remains in the lung, there is some low-level systemic absorption of the drug.

Because of the limitations of calculating pravastatin concentrations in lung tissue (Fig.1) based on estimates of lung density (see Materials and Methods), we also determined the absolute levels of pravastatin (i.e., exact drug concentration) in nanograms per gram of lung tissue *and* in nanograms per right lung. While pravastatin levels were apparently higher in the OVA + i.t. pravastatin group as compared to the FA + i.t. pravastatin group, this difference did not reach statistical significance (14,001 ± 6136 vs. 5221 ± 2439 ng/g; *P* = NS by Mann–Whitney test). There was no detectible pravastatin in the OVA and FA PBS controls, as expected.

Plotted as *ng/right lung*, OVA+i.t. pravastatin had higher levels than FA+i.t. pravastatin, 2637 ± 1823 vs. 1641 ± 858.9, respectively, but this did not reach statistical significance (*P* = NS by Mann–Whitney test). The amount of pravastatin in the right lung is less than the *ng/g* calculation because the right lungs on average weighed 0.243 ± 0.034 g in this experiment.

### Normalized pravastatin levels in OVA- vs. FA-exposed lungs

**Figure 2:**
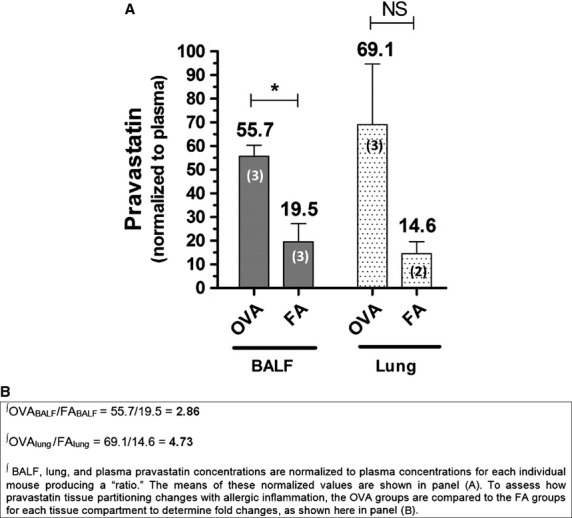
The Effect of Allergic Inflammation on Relative Pravastatin Drug Levels in the Same Tissue Compartments. (A) Pravastatin concentrations for BALF and lung samples were normalized to their respective plasma concentrations for each mouse. In OVA-exposed mice, pravastatin levels in BALF were significantly higher than FA controls (55.7 vs. 19.5, *P = 0.0158 by t test), with a similar trend in homogenized lung tissue (69.1 vs. 14.6, *P* = NS by *t* test). All other relevant comparisons were not statistically significant (*P* = NS by ANOVA or *t* test). (B) OVA groups are compared to the FA groups across each tissue compartment to determine fold change in mean pravastatin levels. Plasma-normalized BALF pravastatin concentration for the OVA group (i.e., OVA_BALF_) was on average 2.86-fold higher than its respective FA_BALF_ group. Plasma-normalized lung pravastatin concentration for the OVA_lung_ group was 4.73-fold higher than its respective FA_lung_ group. The numbers in parentheses listed on each bar graph indicate the number (*n*) of mice per group.

To compare relative pravastatin levels in BALF, lung, and plasma between the FA- and OVA-exposed groups, we first corrected for the relative differences in plasma pravastatin levels by normalizing BALF and lung tissue pravastatin levels to their respective plasma drug concentration for each mouse; we then plotted the means of these normalized values (Fig.2). For plasma comparisons, normalization was not necessary given it was in one tissue compartment. This allowed us to account for potential differences in relative pravastatin absorption from mouse to mouse, thereby allowing comparisons as ratios or normalized values for lung and BALF pravastatin levels.

Normalized BALF pravastatin values were significantly higher in OVA-exposed mice than FA controls (55.7 vs. 19.5, *P = 0.0158 by *t* test). While there was a similar trend in homogenized lung tissue (69.1 vs. 14.6, *P* = NS by *t* test, Fig.2A), these and other relevant comparisons between normalized values were not statistically significant (*P* = NS by ANOVA or *t* test).

We also compared fold changes in pravastatin levels in plasma-normalized lung homogenate and BALF between the FA- and OVA-exposed treatment groups. Fold changes in drug levels due to OVA-induced inflammation gives some indication of the magnitude of pravastatin partitioning when comparing BALF versus lung tissues. The normalized BALF concentration of pravastatin in the OVA-exposed group (OVA_BALF_) was 2.86-fold higher than its respective FA group (FA_BALF_) (Fig.2B). Normalized lung pravastatin concentration in the OVA-exposed group (OVA_lung_) was 4.73-fold higher than its respective FA group (FA_lung_) (Fig.2B). Lung and BALF consistently had the highest relative concentration of pravastatin as compared to plasma, for both OVA and FA groups.

### Pravastatin reduces goblet cell hyperplasia and metaplasia

**Figure 3:**
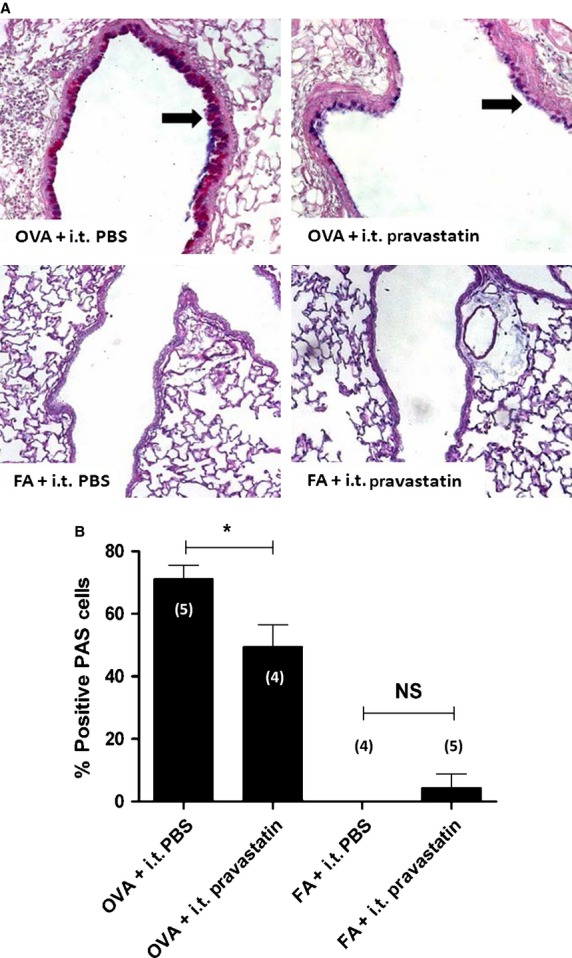
Pravastatin Effect on Goblet Cell Hyperplasia/Metaplasia. Treatment with i.t. pravastatin reduced goblet cell hyperplasia/metaplasia (black arrows, 200× magnification) by 30.5% (*P < 0.05, 1-way ANOVA) in the OVA group. Pravastatin had no significant effect in the FA controls (100× magnification). The numbers in parentheses listed on or above each bar graph indicate the number (*n*) of mice per group.

After establishing that pravastatin was measurable in the lung, we assessed its anti-inflammatory potential and effects on components of airway remodeling. We predicted that the direct application of pravastatin to the airways would attenuate OVA-induced goblet cell metaplasia and hyperplasia. In OVA-exposed mice, PAS staining showed a significant reduction in goblet cell hyperplasia by 30.5% (*P < 0.05, 1-way ANOVA) calculated as “%Positive PAS Cells” in mice treated with i.t. pravastatin compared to mice treated with i.t. PBS. There was no statistically significant change in FA control mice with pravastatin treatment (Fig.3).

### Pravastatin improves airway hypersensitivity but not airway hyperreactivity

Our hypothesis predicted that instilling pravastatin directly into airways would result in potent improvements in airway physiology, that is, inhibition of airway hyperreactivity (AHR) and airway hypersensitivity (AHS), while preserving lung compliance.

**Figure 4:**
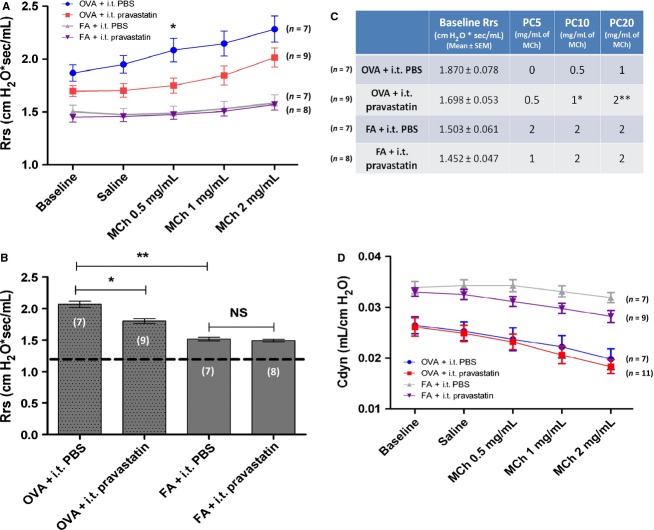
The Effect of Intratracheal Pravastatin on Lung Physiology. (A) Pravastatin had no statistically significant effect on methacholine (MCh)-induced airway hyperreactivity (AHR) except at the 0.5 mg/mL MCh dose (*P < 0.05, by 2-way ANOVA). An independent analysis using linear regression also showed no statistically significant differences (data not shown). (B) Analyzing averaged respiratory system resistance (*R*_rs_) data by treatment group, the OVA + i.t. PBS group had the highest airway resistance (or *R*_rs_) compared to FA + i.t. PBS and FA + i.t. Pravastatin (**P < 0.0001, by Kruskal–Wallis test). Pravastatin reduced the OVA-induced increase in total *R*_rs_ by 14.3% (*P < 0.05, by Kruskal–Wallis test). The horizontal dotted line represents the expected *R*_rs_ for FA controls in our mouse model (see Results section for details). (C) The provocative concentrations (PC) of MCh were higher for OVA + i.t. Pravastatin than OVA + i.t. PBS mice for PC5 (*P* = NS), PC10 (*P = 0.04), and PC20 (**P = 0.024 by Wilcoxon signed rank test). This is consistent with a decrease in airway hypersensitivity (AHS) due to treatment with pravastatin. There were no significant differences in the FA groups. Values for the median MCh dose are listed under the PC5 to PC20 columns. (D) Pravastatin did not improve dynamic lung compliance (Cdyn) (*P* = NS by 2-way ANOVA). The number of mice (*n*) is indicated in parentheses for each treatment group.

We found that pravastatin had differential effects on lung mechanics. While pravastatin inhibited AHS, overall it had no statistically significant effect on AHR. Ovalbumin aerosol exposure increased R_rs_ and induced AHR as compared to FA controls (OVA + i.t. PBS vs. FA + i.t. PBS and vs. FA + i.t. pravastatin; for all three doses of MCh, *P* < 0.0001 by 2-way ANOVA). This indicates an appropriate response to allergen in our model with respect to airway hyperresponsiveness. However, pravastatin had no statistically significant effect on MCh-induced AHR *except* at the 0.5 mg/mL MCh dose (*P < 0.05, by 2-way ANOVA) (Fig.4A). An independent analysis using linear regression to assess change in slope of the *R*_rs_ data showed no statistically significant differences with pravastatin treatment in the OVA groups (*P* = NS, data not shown).

Analyzing total *R*_rs_ data by treatment group, the OVA + i.t. PBS group had the highest airway resistance (or *R*_rs_) compared to FA + i.t. PBS controls (**P < 0.0001, by Kruskal–Wallis test). Administration of i.t. pravastatin reduced the OVA-induced increase in total *R*_rs_ by 14.3% (*P < 0.05, by Kruskal–Wallis) (Fig.4B). Of note, the horizontal dotted line in Fig.4B represents the expected *R*_rs_ for FA controls in our mouse model; it represents a historical average of data from multiple prior experiments using our model system and provides a reference point for the expected *R*_rs_ in FA mice.

To measure pravastatin-dependent changes in AHS, we used the provocative concentrations (PC) of MCh (0.5, 1.0, and 2.0 mg/mL) to cause a 5, 10, and 20% increase from baseline *R*_rs_ (PC5, PC10, and PC20, respectively), representing low, medium, and high MCh doses in our model system (Fig.4C). We used this percentage range because of the constraints of our plethysmograph system in terms of the achievable *R*_rs_ using only three doses of MCh: 0.5, 1.0, and 2.0 mg/mL. For the OVA groups, saline challenge (0 mg/mL MCh) followed by these three doses of MCh increased average R_rs_ above baseline values by a maximum of 18.5 to 22% at the 2.0 mg/mL dose. For the FA groups, this increase ranged from 5.4 to 8.3% at the 2.0 mg/mL MCh dose. We determined that PC5, PC10, and PC20 was an appropriate range of % increase in *R*_rs_ fitting our model system, where PC10 is approximately *half* of the increase in R_rs_ values above baseline. Therefore, within the constraints of our model, PC10 and the range we chose (PC5 to PC20) is a meaningful measure.

The MCh provocative concentrations were higher for pravastatin- than PBS-treated OVA mice for PC10 (*P = 0.04) and PC20 (**P = 0.024 by Wilcoxon signed rank test). For PC10 and PC20 challenges, it takes *twice* the dose of MCh to induce an equivalent % increase in *R*_rs_ in the pravastatin-treated mice as compared to PBS controls. Therefore, treatment with i.t. pravastatin significantly decreased AHS at the medium and high PC doses in OVA-exposed mice. There were no significant differences in the FA groups (*P* = NS, Fig.4C).

In our mouse model, OVA exposure decreases C_dyn_ due to increased airway inflammation, edema, and mucus production. As expected, OVA sensitization and exposure decreased C_dyn_ relative to FA controls (OVA + i.t. PBS vs. FA + i.t. PBS, *P* < 0.001 for MCh doses 0.5 and 1 mg/mL, and *P* < 0.0001 for 2 mg/mL, by 2-way ANOVA. For OVA + i.t. PBS vs. FA + i.t. pravastatin, *P* < 0.05 for MCh doses 0.5 and 1.0 mg/mL, and *P* < 0.01 for dose 2.0 mg/mL, by 2-way ANOVA). While this indicates an appropriate response to allergen exposure in our model with respect to airway physiology, pravastatin had no statistically significant effect on MCh-induced reductions in C_dyn_ (*P* = NS by 2-way ANOVA) (Fig.4D).

### Pravastatin has selective effects on the anti-inflammatory response

We and others have previously shown that giving simvastatin (Zeki et al. 2009) or pravastatin (Imamura et al. 2009) via the intraperitoneal (i.p.) route significantly attenuates systemic and airway Th2 allergic inflammation in OVA models of asthma. We hypothesized that i.t. pravastatin would be more potent at inhibiting Th2 allergic inflammation given the lung-targeted approach. We tested this prediction by examining lung histopathology, BALF total and differential cell counts, BALF cytokine levels, and exhaled NO levels.

**Figure 5:**
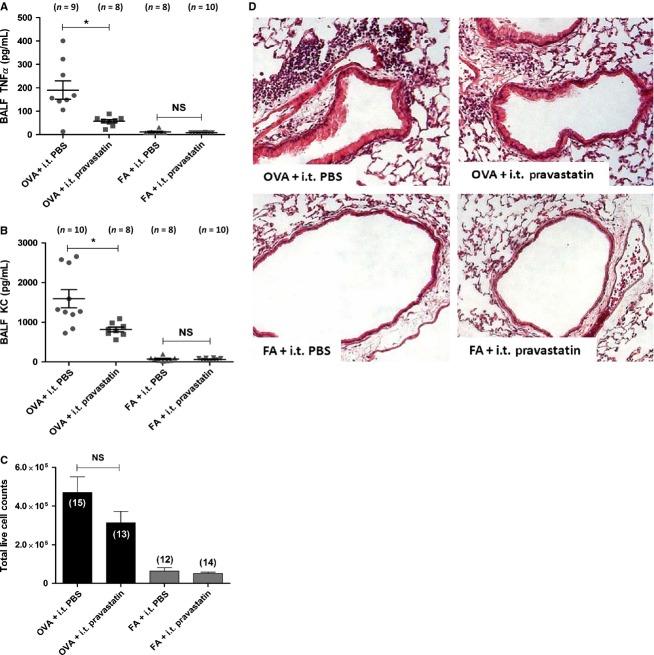
Pravastatin Effect on Lung and Airway Inflammation. (A) Treatment with pravastatin i.t. reduced BALF concentrations of TNF*α* by 60.4% (*P < 0.0001 by 1-way ANOVA) in the OVA groups. (B) Pravastatin reduced BALF concentrations of KC by 48.6% (*P < 0.0001 by 1-way ANOVA) in the OVA groups. (C) Pravastatin i.t. had no statistically significant effect on airway leukocyte influx as measured by BALF total leukocyte (*P* = NS by Kruskal–Wallis test) or differential cell counts (including BALF absolute eosinophil, lymphocyte, macrophage, and neutrophil counts (See Table1)). (D) By qualitative histological assessment on H&E staining, i.t. pravastatin attenuated OVA-induced peribronchiolar inflammation in OVA-exposed mice (200× magnification). There were no visible differences between the FA groups (100× magnification). The number of mice (*n*) is indicated in parentheses for each treatment group.

Although pravastatin decreased levels of TNF*α* and KC in BALF, it did not reduce inflammation in BALF (Fig.5). In the OVA groups, pravastatin decreased TNF*α* by 60.4% (*P < 0.0001 by 1-way ANOVA) and KC by 48.6% (*P < 0.0001 by 1-way ANOVA) in BALF (Fig.5A and B). Pravastatin treatment had no statistically significant effects on other BALF cytokines/chemokines including IL-13, IL-4, eotaxin, RANTES, IL-5, IL-10, IP-10, IFN*γ*, IL-1*α*, IL-1*β*, or IL-17. Of note, MCP-1 was not detectible in any treatment group (by ANOVA or *t* test, data not shown).

Qualitative histological assessment of H&E stained lung sections of pravastatin-treated mice exhibited a reduction in OVA-induced peribronchiolar and lung parenchymal inflammatory cell influx. There were no visible differences in the FA groups with or without pravastatin treatment (Fig.5D). However, pravastatin had no statistically significant effect on the influx of airway leukocytes as measured by BALF total leukocyte counts (*P* = NS by Kruskal–Wallis test) (Fig.5C), or inflammatory differential cell counts (Table1).

**Table 1:** BALF leukocyte differential cell counts

	OVA + i.t. PBS (*n* = 14–16)	OVA + i.t. Pravastatin (*n* = 14–15)	FA + i.t. PBS (*n* = 12)	FA + i.t. Pravastatin (*n* = 12–13)
Abs. Eos. Count	2.9 ± 0.54 × 10^5^	1.9 ± 0.44 × 10^5^	92.9 ± 83.4	730.8 ± 337.9
Abs. Mac. Count	1.5 ± 0.25 × 10^5^	1.3 ± 0.22 × 10^5^	0.59 ± 0.16 × 10^5^	0.49 ± 0.07 × 10^5^
Abs. Lymph. Count	1.7 ± 0.73 × 10^4^	2.0 ± 0.71 × 10^4^	338.8 ± 237.6	286.5 ± 208.3
Abs. Neutr. Count	2.8 ± 1.1 × 10^4^	3.7 ± 1.6 × 10^4^	0.34 ± 0.11 × 10^4^	0.15 ± 0.07 × 10^4^

*P* = NS for all OVA + i.t. PBS vs. OVA + i.t. Pravastatin, and similarly for FA groups.

Abs. (absolute), Eos. (eosinophil), Mac. (macrophage), Lymph. (lymphocyte), and Neutr. (neutrophil).

For all inflammatory cell types (i.e., eosinophil, macrophage, lymphocyte) except for absolute neutrophil count, there was a statistically significant difference in the OVA + i.t. PBS vs. FA + i.t. PBS groups indicating an appropriate allergic inflammatory response in our model (*P* < 0.001, *P* < 0.05, *P* < 0.05, and *P* = NS, respectively). Intratracheal pravastatin had no statistically significant effects in both OVA and FA treatment groups for absolute eosinophil, lymphocyte, and neutrophil counts (*P* = NS by Kruskal–Wallis test), and for absolute macrophage count (*P* = NS by 1-way ANOVA; Table1).

Treatment with pravastatin did not affect FeNO levels among all four treatment groups (OVA + i.t. PBS = 4.3 ± 1.6, OVA + i.t. Pravastatin = 4.3 ± 0.8, FA + i.t. PBS = 3.5 ± 0.5, FA + i.t. Pravastatin = 3.6 ± 0.6 ppb; *P* = NS by 1-way ANOVA). Log transformation of the data and nonparametric analyses also yielded nonsignificant differences (*P* = NS; data not shown).

### Tolerability of intratracheal pravastatin in mice

**Figure 6:**
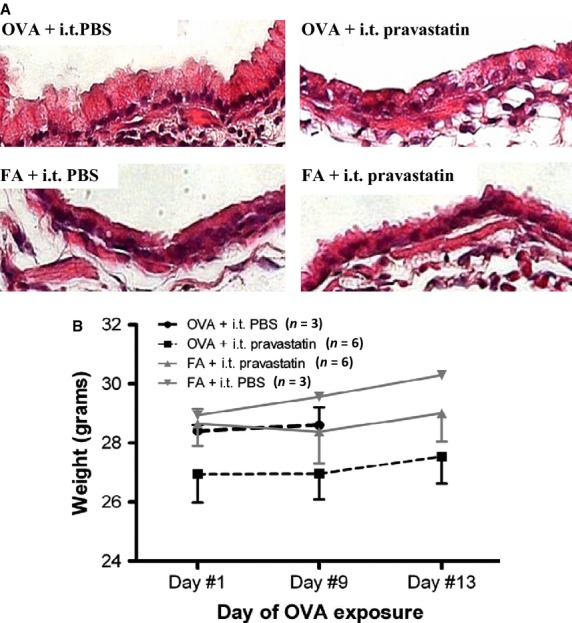
Effect of Intratracheal Pravastatin on Bronchial Epithelium and Mouse Weights. (A) I.t. pravastatin did not damage the airway epithelium as seen by histological assessment (H&E stain, 400× magnification). (B) I.t. pravastatin in both FA controls and OVA-exposed mice was well-tolerated as measured by steady weight gain in all mice (*P *= NS by 2-way ANOVA). There were no statistically significant weight differences between groups on Day #1, that is, at their baseline weights (*P* = NS by 1-way ANOVA). Compared to their own baseline, mice in each group gained weight steadily over time during drug treatment. The number of mice (*n*) is indicated in parentheses for each treatment group.

Intratracheally instilled pravastatin did not appear to induce additional damage to the airway epithelial lining as observed by histological assessment at 400× magnification (Fig.6A). There was no histological evidence of bronchial epithelial denudation or sloughing. Pravastatin in both FA controls and OVA-exposed mice was well-tolerated as measured by steady weight gain in all mice (Fig.6B). There were no statistically significant differences in baseline weights (Day #1) between treatment groups, and compared to their own respective baseline values, all mice in each group gained weight steadily over a period of 1 to 13 days during drug treatment (Fig.6B).

## Discussion

This is the first study that investigates whether administration of pravastatin directly into the lungs mitigates experimental allergic airway inflammation and airway hyperresponsiveness, and whether it is quantifiable in plasma and lung tissues. In this proof-of-principle study, we show that intratracheally instilled pravastatin achieves high concentrations in the lung with low systemic distribution, may have therapeutic potential in asthma, and does not demonstrate acute toxicity in mice. We also developed a novel mass spectrometry method to measure pravastatin in lung tissues allowing us to estimate relative drug distribution. Pravastatin has demonstrated benefits in our model; however, other statins may be more potent via the intratracheal (i.t.) route. Additional research is needed to determine the ideal type of statin and dose to use which can lead to the development of a novel class of inhaler therapy for human asthma.

In our prior work, we established that systemic treatment with simvastatin attenuates allergic inflammation in a MA-dependent manner, decreases AHR, and reduces hallmarks of adverse airway remodeling in animal models of asthma (Zeki et al. 2009, 2010). We also showed that simvastatin directly inhibits IL13-induced expression of proinflammatory cytokines and chemokines including eotaxin, in primary mouse tracheal epithelial cells (Zeki et al. 2012). Using murine models, others have shown that systemic treatment with pravastatin (Imamura et al. 2009) yields similar antiinflammatory effects to simvastatin (Joyce et al. 2001; Yeh and Huang 2004; Krauth et al. 2006; Imamura et al. 2009). We considered whether an airway-targeted approach using pravastatin might also have anti-inflammatory effects while measuring drug levels in different tissue compartments to assess its distribution.

For our experiments we selected pravastatin for two reasons: (1) pravastatin's beneficial anti-inflammatory effect in the ovalbumin mouse model, when given systemically, was shown to be similar to simvastatin; and (2) pravastatin is water soluble, unlike the lipophilic statin simvastatin, and can easily be dissolved in saline for intratracheal instillation, thereby avoiding the use of drug vehicles that could irritate or damage airway mucosa.

We thought that targeting the lung via intratracheal instillation would lower the effective statin doses to achieve similar anti-inflammatory effects in the lung, while reducing systemic absorption and unwanted potential side effects. In some patients, statins can have serious adverse effects including hepatitis and myositis that would preclude their clinical use. Even more common yet milder symptoms of statin use such as myalgias could potentially by bypassed if minimal systemic absorption can be achieved. This might open new therapeutic avenues for pediatric patients with asthma, where statins are not routinely used, or in elderly patients intolerant to statin side effects such as myalgias. Based on biological plausibility (Yeganeh et al. 2014), an inhaled statin may have the potential to mitigate adverse airway remodeling (Murphy et al. 2008; Zeki et al. 2010; Ahmad et al. 2011), in particular goblet cell mucus production (Marin et al. 2013), smooth muscle hyperplasia (Takeda et al. 2006; Schaafsma et al. 2011), subepithelial fibrosis (Watts and Spiteri 2004; Watts et al. 2005; Kim et al. 2010), and extracellular matrix (ECM) production (Li et al. 2008; Schaafsma et al. 2011); where we currently lack adequate therapy.

### Summary of main findings

Pravastatin given by i.t. instillation achieves high relative concentrations in BALF and lung relative to plasma, as measured by mass spectrometry (Fig.1). In comparing the effect of OVA relative to FA controls, pravastatin achieves the highest distribution in lung tissue (greater than BALF or plasma), indicating little but detectible systemic absorption (Fig.2). While pravastatin reduced goblet cell metaplasia/hyperplasia (Fig.3), we saw no statistically significant anti-inflammatory effects besides reductions in select cytokines (TNF*α* and KC) (Fig.5). Importantly, pravastatin reduced AHS and total *R*_rs_, but did not attenuate AHR or preserve lung compliance (Fig.4). Finally, pravastatin administered i.t. was well-tolerated in mice and did not damage the airway epithelium (Fig.6).

There is only one other study that we are aware of which systematically evaluated the impact of simvastatin in murine allergic asthma via multiple routes including by gavage, intraperitoneal injection, intratracheal instillation, and aerosol inhalation; where the authors also reported simvastatin levels in lung and blood (Xu et al. 2012). In this study, they found that intratracheal and inhaled simvastatin had potent anti-inflammatory effects similar to the corticosteroid dexamethasone, and significantly improved both AHR and lung compliance. However, the use of 20% ethanol as simvastatin's drug vehicle raises great concern given the potential cytotoxic effects of alcohol. This high ethanol concentration may preclude use in humans and the rapid translation to a Phase 1 clinical trial, despite the recent development of a novel statin inhaler for human use (Tulbah et al. 2014).

### Novel method for measuring pravastatin in plasma and lung tissues

We used UPLC-MS to measure pravastatin drug concentrations in plasma, lung tissue, and BALF (Fig.1). Although pravastatin has been measured in plasma previously (Jain et al. 2007; Deng et al. 2008; Badolo et al. 2013), the use of mass spectrometry to measure statins in lung tissue has not been previously reported. More specifically, the use of UPLC-MS to quantify statin drug levels, including pravastatin, in lung tissues is novel and unique with respect to the known published literature.

We found that pravastatin remained largely in the BALF and lung tissues, and there are numerous factors that could contribute to this outcome. Because we only examined a single time point after pravastatin i.t. instillation, this observation could be a function of the timing of pravastatin administration relative to the time mice were killed (Fig.1A–C). Specimens were collected at the end of 2-week experiments within a 1 to 2 h window after the last OVA exposure/statin dose. For instance, we did not measure pravastatin in plasma, lung, or BALF at the potential nadir between dosing episodes where the partitioning of pravastatin between these three tissue compartments could have been different (i.e., more equal drug distribution between lung and blood). On the basis of pravastatin plasma concentrations at the prespecified time points (see Materials and Methods), we concluded that minimal yet readily detectible systemic absorption occurred.

Normal lung clearance of the drug may include contributions via the mucociliary escalator, which would result in the cleared drug being swallowed, leading to secondary and indirect systemic administration. Distribution pathways from airways to plasma for pravastatin administered via the intratracheal route could include direct diffusion through the airway endothelium into the bloodstream, lymphatic drainage, or swallowing of lung lining fluid during mucociliary clearance.

Xu et al. (2012) compared intratracheally administered and inhaled simvastatin in their allergic asthma model. The investigators used HPLC to measure simvastatin in different tissue compartments. Similar to our results, they reported much higher simvastatin levels in the lung as compared to plasma measured at 0.5, 2, and 6 h after injection, with the i.t. route achieving much higher levels than aerosol inhalation (186.3 i.t. vs. 6.85 inhaled (*μ*g/g tissue)). This confirms our hypothesis that the majority of the drug remains in the local pulmonary tissue, whereas only a relatively small amount is absorbed systemically at the time range observed.

We also evaluated the effect of allergic inflammation on the partitioning of pravastatin between the plasma, lung, and BALF compartments (Fig.2). After normalizing lung and BALF pravastatin levels to each animal's own plasma pravastatin concentration, we obtained ‘corrected’ or normalized relative pravastatin values (see Materials and Methods and Results). This allowed us to correct for any potential variation from mouse to mouse in relative systemic absorption of the drug. Then we compared these normalized pravastatin levels as ratios of OVA-to-FA, and observed that during OVA-induced inflammation, pravastatin levels are 4.73-fold higher in the lung but only 2.86-fold higher in BALF as compared to FA controls; and with stable levels in plasma (Fig.2B).

These data suggest that during inflammation, more pravastatin enters (or remains) in the lung tissue as compared to BALF. We speculate that under conditions of allergic inflammation, epithelial-vascular leaking may underlie the higher pravastatin drug levels found in the lung compared to BALF. Surprisingly, plasma drug levels between OVA and FA groups did not change by comparison of either drug concentrations (Fig.1B and C) or normalized ratios (see Results section). Despite a higher drug concentration in the lung as compared to BALF or plasma, pravastatin's lack of significant anti-inflammatory effects on leukocyte influx is unexpected.

### Pravastatin effects on airway epithelial remodeling

Pravastatin's most potent effect was on reducing airway goblet cell metaplasia/hyperplasia (Fig.3). Direct instillation of pravastatin into mouse airways attenuated OVA-induced goblet cell hyperplasia by approximately one third, an effect that is comparable to that seen with systemic simvastatin treatment (Kim et al. 2007; Zeki et al. 2010). This finding is not surprising given that statins are known to have direct effects on airway epithelial cells (Sakoda et al. 2006; Murphy et al. 2008; Takahashi et al. 2008; Planagumà et al. 2010; Xing et al. 2011; Iwata et al. 2012; Zeki et al. 2012; Brandelius et al. 2013; Lee et al. 2013), including simvastatin which reduces mucus production in Calu-3, a human airway epithelial cell line (Marin et al. 2013). In vivo application of inhaled simvastatin also reduced airway goblet cell remodeling and mucus production (Xu et al. 2012) similar to our results with i.t. pravastatin (Fig.3).

The Muc5AC gene is one of several key mucin genes that programs epithelial cell goblet cell differentiation and mucin production. Simvastatin treatment by the intraperitoneal route reduces Muc5AC mRNA in rat lungs (Ou et al. 2008). Similarly, intragastric administration of simvastatin also reduces Muc5AC mucin synthesis at both the mRNA and protein synthesis levels in rat lungs (Chen et al. 2010).

Whether statins have direct effects on Muc5AC gene expression remains an open question. Because IL13 induces Muc5AC expression in human airway epithelial cells (Zhao et al. 2009), and statins are known to inhibit IL13 production in lungs (Zeki et al. 2009), we speculate that this could be one potential mechanism of how statins attenuate mucin production in airways. Because overproduction of mucus in humans is found in 80% of lethal cases of severe asthma, pravastatin's direct effect on goblet cells is evidence supporting the potential benefits of developing statin inhalers.

Pravastatin inhibition of goblet cell hyperplasia/metaplasia may be independent of inflammation given that pravastatin did not inhibit airway leukocyte influx in BALF to a significant degree. We speculate that pravastatin's beneficial effects via the i.t. route may be predominantly limited to the airway epithelial lining at the dose used (30 mg/kg). Additional research is needed to examine whether or not this is a sustained phenomenon or something related to our experimental protocol, dosing, and timing of drug administration.

### Pravastatin effects on inflammation

Despite the fact that i.t. administration of pravastatin achieved appreciably high concentrations in BALF and lung tissue (Figs.1, 2), pravastatin did not consistently reduce inflammatory cell influx as measured in BALF. Although we observed reduced peribronchiolar inflammation by H&E staining (Fig.5D) and inhibition of TNF*α* and KC in BALF (Fig.5A and B), the lack of statistically significant anti-inflammatory effects of pravastatin on BALF cell counts (Fig.5C and Table1) was a surprise given what is known about statins in general, and what we know about pravastatin in the OVA model (Yeh and Huang 2004; Imamura et al. 2009). Furthermore, it remains unclear whether i.t. pravastatin affects inflammatory cell migration from the peribronchial region into the airway lumen or tissue adherence which may affect the visual impressions of our lung histology results as seen in Fig.5D.

There may be several reasons for this unexpected result. First, unlike the more lipophilic statins such as simvastatin, fluvastatin, and atorvastatin; pravastatin is the most hydrophilic statin available and therefore does not enter cells via passive diffusion. Therefore, it is possible that while pravastatin is in the lung tissue, it is unable to efficiently enter airway resident cells such as the epithelium or inflammatory/immune cells. Pravastatin requires specific organic anion transporting polypeptides in order to pass through the cell plasma membrane, and it remains unknown if lung or airway cells express these transporters (see below). Second, pravastatin's effect could have been short lived and therefore missed during the time points we evaluated. Third, pravastatin's beneficial effects with respect to anti-inflammatory potency may be limited to immune cell and endothelial cell functions, rather than to airway epithelial or mesenchymal cells. Fourth, the concentration we used may not have been sufficient to inhibit inflammation; however, the pravastatin dose we used (30 mg/kg) is the maximal concentration where the drug remains soluble in aqueous solutions. Fifth, pravastatin may be more effective via systemic routes (i.e., i.p. or oral) (Yeh and Huang 2004; Imamura et al. 2009), rather than via inhalation or direct airway instillation. And finally, it is possible that a chronic OVA exposure model (i.e., 8 or 10 weeks) might yield different results with respect to anti-inflammatory and antifibrotic effects of pravastatin. Additional investigations are needed in order to answer these important questions.

### Pravastatin effects on pulmonary mechanics

On the basis of known beneficial effects of statins on lung physiology (Chiba et al. 2008a,b; Zeki et al. 2009; Cazzola et al. 2011; Xu et al. 2012), we predicted that i.t. pravastatin would have potent effects on airway resistance and lung compliance. Instead, we found that pravastatin had differential effects on airway mechanics. Despite reducing total *R*_rs_ (Fig.4B) in OVA-exposed mice, pravastatin overall did not improve lung compliance or attenuate MCh-induced AHR (Fig.4A and D).

However, treatment with i.t. pravastatin did reduce AHS, a clinically relevant component of airway hyperresponsiveness. Pravastatin-treated animals required MCh doses twice as high as the PBS control group to achieve an equivalent increase in *R*_rs_ above baseline (Fig.4C). These results indicate that pravastatin's protective effect on airway resistance is limited to the sensitivity of airways to broncho-constricting agents. This translates to higher broncho-provocating MCh doses needed to achieve a given increase in *R*_rs_ in statin treated mice, indicating a protective effect of pravastatin. However, once this sensitivity threshold is surpassed then pravastatin offers no additional mitigating effects on AHR or bronchospasm (O'Byrne and Inman 2003; Affonce and Lutchen 2006; Turi et al. 2011).

There are several potential reasons for this uncoupling of AHR and AHS effects due to pravastatin treatment. It may be that pravastatin did not reach airway smooth muscles and instead remained on the luminal epithelial side or only within the epithelial cells, thereby having no effect on AHR. Another potential explanation is direct effects on airway neuronal cells or their conductive function (Tarhzaoui et al. 2009), thereby affecting the neural contribution to bronchoconstriction. Of note, different agonists or pharmacologic agents can induce changes in both AHR and AHS. However, they can also affect either AHR or AHS independently, yielding specific effects on only one component of airways hyperresponsiveness (Lötvall et al. 1998; Chapman et al. 2014).

### Statin physiochemical properties and their anti-inflammatory effects

The physiochemical properties of statins may have significant effects on the absorption and activation of statins in vivo, determining both transport inside cells and their conversion to the hydroxy acid, the active form of statin that binds HMGCR (Hamelin and Turgeon 1998; Istvan and Deisenhofer 2001). Statin polarity (i.e., hydrophilicity vs. hydrophobicity) may impact statin tissue absorption and distribution, and thereby its ultimate anti-inflammatory potential.

Simvastatin is the most lipophilic of the statins used clinically, whereas pravastatin is the most hydrophilic. While these properties of polarity inform experimental design with respect to drug solubility and delivery, they may be equally pertinent for cell penetration, and thus, statin bioavailability, potency, and efficacy. The active form of statins is the ‘open ring’ hydroxy acid (e.g., simvastatin acid) which binds to the active site of HMGCR to inhibit its enzymatic function. Simvastatin is administered as an inactive prodrug (‘closed ring’ lactone). Enzymes such as serum lactonases and paraoxonases (Draganov et al. 2000), alkaline hydrolases (Hamelin and Turgeon 1998), and carboxylesterases open the ring to produce the active form of simvastatin. These enzymes also hydrolyze other statins besides simvastatin.

Unlike simvastatin, pravastatin is administered as the active hydroxy acid. However, this active form of statins is not as readily absorbed by cells as is the inactive form. Intracellular uptake of pravastatin requires organic anion transporting polypeptide (OATP) cell membrane transporters which are variably found in peripheral tissues outside the liver. Whether OATPs are expressed in normal human lungs and specifically airway epithelial cells is not known. By comparison, simvastatin enters cells by passive cell membrane diffusion given its lipophilicity, and is more likely than pravastatin to enter the systemic circulation and reach extra-hepatic tissues (Kleemann and Kooistra 2005).

Among the statins, pravastatin is the most hydrophilic with a partition coefficient (Log D) of −0.84 at a pH of 7.4 (Mctaggart 2003) (or −0.23 at a pH of 7.0) (Serajuddin et al. 1991). Although the hydrophilicity of oral pravastatin allows for relatively high circulating concentrations, it also hinders penetration to peripheral tissues and prevents passive diffusion into cells (Hamelin and Turgeon 1998; Vaughan and Gotto 2004).

Cellular uptake of pravastatin is dependent on carrier-mediated transport proteins, primarily OATP2 and OATP1B1 (a.k.a. OATP-C) in humans, which are abundant in hepatocytes but not known to be expressed in lungs. However, there is variable expression in other peripheral tissues such as human brain, kidney, liver, intestines, testis, placenta, heart, and skin (Hsiang et al. 1999; Cheng et al. 2005; Kalliokoski and Niemi 2009). Other pravastatin transporters include OATP1B3 (Seithel et al. 2007) and OATP2B1 (Nozawa et al. 2004; Kalliokoski and Niemi 2009). Mouse OATP expression is highly variable in different tissues, but is expressed in mouse lungs (OATP2a1, OATP3a1, OATP4c1, OATP5a1) (Cheng et al. 2005); however, it remains unknown whether these specific forms of OATP transport pravastatin. Rat OATP1 and OATP2 transport pravastatin, but they are not expressed in lung tissue (Noe et al. 1997; Hsiang et al. 1999; Hasegawa et al. 2010). Whereas, OATP3 is expressed in lung tissue in rats, but there is no report of pravastatin transport capability (Walters et al. 2000; Ohtsuki et al. 2004).

Therefore, while OATPs are expressed at relatively high levels in murine lungs, we do not know whether they can transport pravastatin across the epithelial cell membrane. Although pravastatin was administered directly to the airways in our studies, the lack of a mechanism to readily enter cells may have prevented pravastatin from reaching high enough intracellular concentrations to inhibit HMGCR by any appreciable amount, thus providing little to no therapeutic anti-inflammatory effect, despite beneficial effects on goblet cells and AHS. However, our experimental design also does not exclude other potential pleiotropic effects of pravastatin. Future studies should measure the expression of OATPs in lungs, including in airway epithelial cells, in order to test this hypothesis.

Conversely, simvastatin is 173 times more lipophilic than pravastatin (Log P of 4.68) in the lactone form, and 195 times more lipophilic than pravastatin in the hydroxy acid form (at a pH of 7.0) (Serajuddin et al. 1991), and is therefore capable of passively diffusing through cell membranes without the need for anionic transporters (Serajuddin et al. 1991; Hamelin and Turgeon 1998; Schachter 2005). However, the bioavailability of oral simvastatin is only 5%, primarily due to low solubility (95–98% protein bound) and an efficient first-pass metabolism (Corsini et al. 1999; Vaughan and Gotto 2004; Schachter 2005; Pandya et al. 2008). By bypassing this first-pass metabolism through the administration of low dose simvastatin via the intratracheal route, potent anti-inflammatory effects in the airways are observed (Xu et al. 2012). Passive diffusion may allow intracellular simvastatin levels to reach high enough concentrations to provide the myriad of beneficial effects predicted but not observed in our study using pravastatin.

The ability to passively diffuse through cell membranes may also allow simvastatin to escape from lung tissue to the systemic circulation at a higher rate than pravastatin. This might prevent potential statin-induced toxicities caused by an over-accumulation of the drug in lung tissue. However, higher systemic absorption of simvastatin compared to pravastatin may also result in higher systemic side effects. Xu et al. (2012) did not report any toxicity or adverse reactions with i.t. or inhaled simvastatin (using 20% ethanol as drug vehicle). By comparison, the inability of pravastatin to freely cross cell membranes may also inhibit it from exiting the lung compartment to enter the systemic circulation, potentially allowing the drug to concentrate in the airways or lung tissue and increase the risk of potential statin-induced toxicities (Schachter 2005). In our experiments, we did not observe any pravastatin related lung or systemic toxic effects (Fig.6). Additional research is required to answer these important questions given their therapeutic and safety implications.

We speculate that while i.t. pravastatin is a logical and pragmatic choice given its translational potential in human asthma, its physiochemical properties likely contributed to low intracellular levels despite being found in high concentrations in lung tissue homogenate and BALF. While it is possible that the pulmonary anti-inflammatory effect of statins may be due to systemic (Imamura et al. 2009; Zeki et al. 2009) rather than localized effects in the lung, the work by Xu et al. using simvastatin does not support this idea. In our experiments, low-level systemic absorption of pravastatin was not adequate enough to significantly reduce lung or airway inflammation (Figs.1, 5). However, a different and more lipophilic statin such as simvastatin could potentially produce the desired effects via inhalation in human asthma.

### Alternative methods of administering statins

At present, the only approved route of statin drug administration in humans is through oral ingestion. There are many benefits to administering statins orally, such as ease of administration, good patient compliance, minimal sterility constraints, and cost-effectiveness. However, there are also some drawbacks to oral administration. For example, the bioavailability and the general circulation of statins are relatively low due to first-pass metabolism and protein binding (Hamelin and Turgeon 1998; Vaughan and Gotto 2004). This in part depends on the extent of binding to plasma proteins and cell permeability, and these factors depend on the physiochemical properties of the statin in question. Research is needed to develop better and more efficient ways to administer statin drugs that can increase their bioavailability in specific tissue compartments or organs, such as the airways or lungs.

Other potential routes for statin delivery include nasal (Jha et al. 2014), intravenous (Prinz et al. 2008), subcutaneous (Bews et al. 2014; Jha et al. 2014), intratracheal nanoparticle (Chen et al. 2011), nebulized formulations for direct inhalation, and controlled-release statin-loaded microspheres (Kanjickal et al. 2004; Vishwanathan 2008). Nanocarriers are also a potential innovative drug vehicle, and in particular, novel formulations that can deposit and concentrate in the lung before releasing their statin payload in order to achieve higher local steady-state drug levels.

Our study is the first to evaluate the potential of i.t. pravastatin in asthma and the second to evaluate statin administration via the intratracheal route (Xu et al. 2012). There are several potential benefits of administering statins via inhalation and a rationale to support this therapeutic approach: Lower effective dose as compared to systemic treatment, reduced systemic absorption with attendant lower systemic side effects, direct effect on airway epithelial cells, potential treatment for airway smooth muscle hypertrophy/hyperplasia, potential use in pediatric patients, an alternative option for patients for whom oral statins are contraindicated due to myopathy or other adverse effects, and combination therapy with other standard-of-care inhaler medications, such as ICS, LABA, LAMA.

### Study limitations

There are several limitations to our study. While plasma and BALF pravastatin concentrations were directly measured and quantified in units of ng/*μ*L, lung pravastatin concentration was estimated based on an approximation of lung density. This allowed for relative comparisons to be made across three major tissue compartments. However, our results should be interpreted with this limitation in mind; these are, at best, relative comparisons. Additional calculations using rat lung densities gave us similar results (data not shown). We therefore determined that an estimate of lung pravastatin concentration is a reasonable approximation for the necessary comparisons to BALF and plasma pravastatin levels. In the Results section, we also report the exact drug concentration per lung and per gram of lung.

A given volume of plasma, BALF, and lung tissue may not be the same with respect to the distribution of pravastatin, or any drug for that matter. However, because pravastatin is highly water soluble, we assumed that pravastatin was in equilibrium with total body water. If this assumption is true, we can then directly compare pravastatin concentrations between all three tissue compartments (in units of ng/*μ*L, Fig.1). Therefore, any conclusions drawn from these data should be tempered by this potential limitation.

We did not measure pravastatin concentrations at different time points, thereby excluding the nadir levels on days between statin treatments. This could have yielded different results with respect to the equilibrium achieved between the three tissue compartments; BALF, lung, and plasma. Thus, it is possible that relative pravastatin levels would have differed from what we observed in Figs.1, 2. However, given similar findings by Xu et al. using simvastatin at earlier time points (Xu et al. 2012), we believe our results are valid and pertinent.

With respect to pravastatin's effect on lung physiology, we recognize that our use of invasive plethysmography yields limited information as compared to the more sensitive forced oscillation technique (FOT). While we were able to detect statin-mediated beneficial effects on AHS, other more specific effects on airway narrowing, peripheral tissue resistance, and tissue elastance typically assessed using FOT were not measured using our technique. Therefore, it is possible that additional statin effects on airway or lung function remain undiscovered. Future work in this field using FOT could yield additional important results worthy of further study.

## Conclusions

Our data demonstrate the ability to directly measure and detect pravastatin in lung tissues using mass spectrometry, and the ability of the drug to concentrate in the lung while achieving minimal systemic absorption. Our results also indicate that intratracheal pravastatin has the potential to mitigate some pathological features of experimental allergic asthma. While anti-inflammatory effects were modest at best, i.t. pravastatin reduced the production of select cytokines, attenuated goblet cell epithelial remodeling, and reduced AHS. Finally, pravastatin applied directly to airways in vivo appeared to be safe and well-tolerated by mice.

The use of UPLC-MS allowed us to measure statins in any tissue, in particular the lungs. This will be an important aspect of translating findings from animal models to humans, as the need to quantify statins in varied tissues remains relevant in the human host as we explore alternative routes of statin delivery.

Our results indicate that the statins should be explored as a potential novel class of inhaler therapy for airway diseases such as asthma. However, we believe that additional preclinical work is needed to determine the optimal statin dose, route of administration (and safety of inhalation long-term), and mechanism(s) involved in order to guide future translational studies. We predict that statins with greater lipophilicity may yield more potent anti-inflammatory effects than pravastatin. Work in this area of “lung-directed” or inhaled statins is a new chapter in the evolution of these drugs for the treatment of lung diseases.

## Conflict of Interest

None declared.
